# Chinese Medicine's Intervention Effect on Nogo-A/NgR

**DOI:** 10.1155/2012/528482

**Published:** 2011-12-19

**Authors:** Xiu-de Qin, Li-yuan Kang, Yu Liu, Yan Huang, Shuo Wang, Jin-qiang Zhu

**Affiliations:** ^1^Institute of Traditional Chinese Medicine, Tianjin University of Traditional Chinese Medicine, No. 312 Anshanxi Road, Nankai District, Tianjin 300193, China; ^2^The Second Clinical Medical School, Guangzhou University of Chinese Medicine, Guangzhou 510405, China; ^3^Encephalopathy Center, Guangdong Hospital of TCM, Guangzhou 510120, China

## Abstract

Cerebral vascular disease is very common in the elderly and is one of the most dangerous diseases which is hazardous to the body's health, and it is the medical specialists' study hot spot not only in the clinical field but also in the medical basic research field. Neural regeneration has been paid more and more attention in recent years. Nogo's function in the process of neural regeneration has become the focal point since it was discovered in the year 2000. Many studies elucidate that Nogo negatively affects the neural regeneration and plasticity. Chinese medicine plays an important role in the prevention and treatment of neural diseases, and recently some researches about the Chinese medicine's intervention effect on Nogo-A/NgR sprang up, so it is necessary to make a review on this aspect.

## 1. Nogo's Discovery

That Nogo gene was successfully cloned in 2000 is one of the most imperative breakthroughs since the neural regeneration inhibitors' discovery. The Nogo isoforms A, B, and C are members of the reticulon family of proteins. Nogo-A and Nogo-C are highly expressed in the central nervous system, with Nogo-C being additionally found in skeletal muscle, whereas Nogo-B is found in most tissues. Of the three major isoforms of Nogo, Nogo-A is the most intense inhibitor of neural regeneration in central nervous system. It can inhibit axonal growth both in vitro and in vivo, remarkably [1, 2]. 

## 2. Nogo-A/NgR's Distribution

### 2.1. Nogo-A/NgR's Distribution in the Body

Nogo-A/NgR is widely distributed in the nervous system, but seldomly in the viscus. In human fetal tissue, Nogo-A is strongly expressed in the two-thirds of the ventral of the spinal cord, the dorsal root ganglia, and autonomic ganglia. Similarly, Nogo-A mRNA expression is observed in the adult human spinal cord and ganglia. High levels of Nogo-A message are observed in motor neurons and sensory ganglia neurons. In addition, expression of Nogo-A mRNA is observed in developing muscle tissue. In fetal rats, the adrenal gland and cell clusters in the liver were positive for the Nogo-ABC pan-probe, but negative for the Nogo-A probe [3]. Throughout much of the adult central nervous system (CNS), Nogo-A is detected in oligodendrocyte processes surrounding myelinated axons, including areas of axon-oligodendrocyte contact. Nogo-A receptor (NgR) expression is restricted to postnatal neurons and their axons. In contrast, Nogo-A is observed in myelinating oligodendrocytes, embryonic muscle, and neurons. After spinal cord is injured, Nogo-A is upregulated to a moderate degree, whereas NgR levels are maintained at constant levels. Taken together, these data confirm the apposition of Nogo ligand and NgR in situations of limited axonal regeneration and support the hypothesis that this system regulates CNS axonal plasticity and recovery from injury [4]. 

### 2.2. Nogo-A/NgR's Distribution in the Brain

Study indicated that neurons in the adult rat brain were generally positive, and very prominent nogo-A mRNA and nogo-ABC mRNA signals were obtained from neurons of the hippocampus, piriform cortex, the red nucleus, and the oculomotor nucleus. Nogo mRNA was expressed in neurons and oligodendrocytes, but not astrocytes or Schwann cells [3]. *In situ* hybridization method was used to investigate the expression of mRNA for NgR in unoperated adult rats and mice. NgR was strongly expressed in neurons of the neocortex, hippocampal formation, amygdaloid nuclei, and dorsal thalamus and moderately expressed in the red nucleus and vestibular nuclei. NgR mRNA was expressed in cerebellar deep nuclei and more strongly in granule cells than in Purkinje cells. Large regions of the forebrain, including the striatum, thalamic reticular nucleus, hypothalamus, and basal forebrain showed little or no NgR expression. Nerve implantation into the brain did not affect NgR expression. Some regeneration-competent neurons expressed NgR but others did not. Nogo-66 transcripts were strongly expressed in many classes of CNS neurons and less strongly in white matter [5]. 

## 3. Nogo-A/NgR's Biological Function and Adhibition

### 3.1. Nogo-A/NgR's Main Biological Function

Nogo-A/NgR's function has been extensively explored in recent years, and their main physiological function is believed to be maintaining the stabilization of nervous system and regulating the plastic rearrangements and regeneration after neural injury. An interaction of Nogo on the oligodendrocyte surface with NgR on axons has been suggested to play an important role in limiting axonal growth [4]. Nogo-A and NgR are potent neurite growth inhibitors in vitro and play a role in inhibition of axonal regeneration following injury and central nervous system structural plasticity in vertebrates [6, 7]. The role of Nogo-A in limiting axonal fiber growth and regeneration following the injury of the mammalian CNS is well known, The present results show a unique role of Nogo-A expressed in the adult hippocampus in restricting physiological synaptic plasticity on a very fast time scale. Nogo-A could thus serve as an important negative regulator of functional and structural plasticity in mature neuronal networks [8]. Nogo-A plays a major role in stabilizing and maintaining the architecture of hippocampal pyramidal neurons; although the majority of the activity of Nogo-A relies on a receptor-mediated mechanism involving NgR1, its cell-autonomous function plays a minor role [9]. Nogo could affect memory ability through regulating synapse remodeling. Nogo-A is present in inhibitory presynaptic terminals in cerebellar Purkinje cells at the time of Purkinje cell-deep cerebellar nuclei inhibitory synapse formation and is then downregulated during synapse maturation [10]. NgR1 participates in ligand-dependent inhibition of synaptic plasticity. Loss of NgR1 leads to increased phosphorylation of extracellular signal-regulated kinase 1/2, signaling intermediates known to regulate neuronal growth and synaptic function [11]. NgR might have wider effects on inflammation in a variety of neurological conditions ranging from central nervous system trauma to diseases such as multiple sclerosis or Alzheimer's disease [12].

### 3.2. Main Studies of the Adhibition of Nogo-A/NgR in Neural Disease

#### 3.2.1. In Neurological Deficits

One study indicated that improvement of chronic neurological deficits and enhancement of neuronal plasticity can be induced in the adult rat with anti-Nogo-A immunotherapy, and that this therapy may be used to restore function even when administered long after ischemic brain damage has occurred [13]. Karlén et al.'s experiment showed that mice with inducible overexpression of NgR1 in forebrain neurons have normal long-term potentiation and normal 24 h memory, but severely impaired month-long memory in both passive avoidance and swim maze tests, while blocking transgene expression normalizes the memory impairments [14].

#### 3.2.2. In Cerebral Ischemia

Blocking or refraining Nogo-A pathway has displayed promising effect on cerebral ischemia. Wiessner et al. [15] used monoclonal anti-Nogo-A antibody (7B12) to treat cerebral ischemia rats successfully and considered that specific anti-Nogo-A antibodies bear potential as a new rehabilitative treatment approach for ischemic stroke with a prolonged time-to-treatment window. Chun-Mei et al.'s study [16] indicated that scalp site medication injection could stimulate endogenous neural stem cells proliferation and promote their differentiation and also could inhibit the expression of the nerve regeneration inhibitory factor Nogo-A in brain, there by promoting the repair of brain.

#### 3.2.3. In Traumatic Brain Injury

Functional recovery is markedly restricted following traumatic brain injury (TBI), partly due to myelin-associated inhibitors including Nogo-A, myelin-associated glycoprotein, and oligodendrocyte myelin glycoprotein, which are all bound to the NgR1. Hånell et al.'s study indicated that cognitive function (as evaluated with the Morris water maze at 4 weeks after injury) was significantly impaired both in NgR1 −/− mice and in mice treated with soluble NgR1. In the sNgR1 study, they evaluated hippocampal mossy fiber sprouting using the Timm stain and found it to be increased at 5 weeks following TBI. Neutralization of NgR1 significantly increased mossy fiber sprouting in sham-injured animals, but not in brain-injured animals. And their study urge caution when inhibiting NgR1 in the TBI's early post injury period [17].

#### 3.2.4. In Injured Spinal Cord

Nogo-neutralizing antibody IN-1 increases c-Jun protein levels and protects the injured spinal cord by inhibiting c-Fos protein levels. Moreover, the effects of IN-1 combined with neurotrophin-3 are more significant than with IN-1 alone [18].

#### 3.2.5. In Alzheimer Disease

Nogo-A upregulation and Nogo receptor downregulation in AD rats have relationship with the pathological changes. The estrogen can normalize the expressions of Nogo-A and Nogo receptor so that estrogen can protect neurons from damages. The mechanism of Nogo-A upregulation and Nogo receptor downregulation induced by estrogen in AD models needs to be further researched. Nogo-A and Nogo receptor are presumed to be involved in the pathological processes of AD [19].

## 4. Chinese Medicine's Intervention on Nogo-A/NgR

### 4.1. In Cerebral Ischemia

Studies have manifested that some Chinese medicine showed good effect on cerebral ischemia through regulating the expression of Nogo-A. Huai-Qiang et al.'s study [20] indicated that Nogo-A expression was observed at a short period after cerebral infarction in the model group and increased gradually, reaching a peak at 2 weeks and was still positive at 6 weeks after infarction. The differences between model and normal control groups were significant at the same stage (*P* < 0.01). The difference between Fujian tablet group and model group was not significant (*P* > 0.05) at 3 days, but significant (*P* < 0.01) at other stages; thus demonstrating that Fujian tablet can promote neurogenesis by inhibiting the expression of Nogo-A. Fujian tablet could also inhibit the expression of Nogo-A mRNA in cervical spinal cords of middle cerebral artery occlusion (MCAO) rats, which facilitates regeneration and remodeling of cervical spinal cords [21]. The number of Nogo-A-immunoreactive cells at the 3rd day after reperfusion in panaxtrial saponins (PTS) group decreased compared with the placebo group (*P* > 0.05). And the number at the 7th day in PTS group decreased markedly and represented significant difference compared with the placebo group (*P* < 0.05). This study implies that PTS could downregulate the expression of Nogo-A, and it might be one of the protective mechanisms of PTS against ischemia-reperfusion injury [22]. Wen-Tao et al. duplicated chronic ischemic mice model and observed the butylphthalide's intervention effect on the expression of Nogo-A; they found that the expression of Nogo-A in the low dose and high-dose especially high-dose butylphthalide group was obviously reduced than the model group, thus indicating that butylphthalide's protective effect on chronic ischemic is probably through downregulating the expression of Nogo-A [23]. Wang's experiment manifested that compound Shouwuxianhai tablet could remarkable restrain the Nogo-A expression in MCAO rat brain, therefrom promoted the neural regeneration after ischemic stroke [24].

### 4.2. In Hypoxic-Ischemic Brain Damage

Altered Nogo-A expression was associated with inversely altered synaptophysin expression. The use of ephedrine normalized expression levels of Nogo-A and synaptophysin following hypoxic-ischemic brain damage (HIBD). Chen et al.'s study demonstrated that Nogo-A expression was significantly reduced in the ephedrine group compared with the HIBD group (*P* < 0.01). Synaptophysin expression was significantly decreased in the hypoxic-ischemic cortex, compared with the sham operation group (*P* < 0.01). Synaptophysin levels were significantly increased in the ephedrine group, compared with the HIBD group (*P* < 0.01) [25]. 

### 4.3. In Situation of Injured Spinal Cord

Chinese Medicine Sui fukang (SFK) is possible to improve microenvironment in the injured spinal cord and to make nervous tissue regenerate through restraining the expression of Nogo-A. Animal experiment indicated that the positive cells of Nogo-A mRNA were more and their OD value was higher in the model group than those in other groups (*P* < 0.05); the Nogo-A mRNA positive cells were less and their OD value was lower in SFK group than those in the model group (*P* < 0.05). The changes of Nogo-A protein positive cells number and its OD value were similar with those of Nogo-A mRNA [26]. 

## 5. Problem and Prospect

Neuroscience has stepped forward a lot for the past few years, and more and more attention is paid to the neuro regeneration and neuroremodeling. As one of the most intensive neuroregeneration inhibitors, Nogo-A has been focused on a lot since it was discovered. Many ways related to Nogo-A were used to promote the regeneration of neurons in neural disease, such as Nogo's inhibitor, Nogo' antibody, knockout of Nogo gene, and any other possible medication which could refrain the expression of Nogo. 

Stroke and dementia are closely associated, whether in the form of vascular cognitive impairment or Alzheimer's disease. At least 35 million people worldwide currently have dementia. Dementia prevalence is predicted to double every 20 years: an expected 66 million in 2030 and 115 million in 2050. The burden of these diseases is considerable when taken with the annual 15 million people worldwide who will suffer stroke [27]. It is well known that Chinese medicine could take effect through multiple target points, and it is believed that Chinese medicine has good effect on neural diseases, especially on the treatment of stroke, dementia, and other chronic neural degeneration diseases. There are already some well-known Chinese medicine agents which have potential good therapeutic effect on stroke or dementia patients, such as Ginkgo Biloba extract, Lamps spend grain injection, and Huperzine A. Chairman MAO once said: “Chinese medicine is a great treasure and we should make effort to explore it well”, so the step of developing more effective Chinese patent drug from Chinese medicine should never stop, and whether are there any Chinese medicine which could take effect on stroke or dementia through regulating the Nogo-A/NgR level or signal transduction pathway should be further studied.
